# Adiponectin Alleviates Genioglossal Mitochondrial Dysfunction in Rats Exposed to Intermittent Hypoxia

**DOI:** 10.1371/journal.pone.0109284

**Published:** 2014-10-20

**Authors:** Hanpeng Huang, Xiufeng Jiang, Yanbin Dong, Xiaofeng Zhang, Ning Ding, Jiannan Liu, Sean Z. Hutchinson, Gan Lu, Xilong Zhang

**Affiliations:** 1 Department of Respirology, Affiliated Hospital of Jiangsu University, Zhenjiang, China; 2 Department of Respirology, Nanjing Medical University affiliated Wuxi People’s Hospital, Wuxi, China; 3 Department of Respirology, The First Affiliated Hospital with Nanjing Medical University, Nanjing, China; 4 Department of Respiratory Diseases, Jangsu Geriatric Hospital, Nanjing, China; 5 Morsani College of Medicine, Tampa, FL, United States of America; Virgen Macarena University Hospital, School of Medicine, University of Seville, Spain

## Abstract

**Background:**

Genioglossal dysfunction is involved in the pathophysiology of obstructive sleep apnea hypoxia syndrome (OSAHS) characterized by nocturnal chronic intermittent hypoxia (CIH). The pathophysiology of genioglossal dysfunction and possible targeted pharmacotherapy for alleviation of genioglossal injury in CIH require further investigation.

**Methodology/Principal Findings:**

Rats in the control group were exposed to normal air, while rats in the CIH group and CIH+adiponectin (AD) group were exposed to the same CIH condition (CIH 8 hr/day for 5 successive weeks). Furthermore, rats in CIH+AD group were administrated intravenous AD supplementation at the dosage of 10 µg, twice a week for 5 consecutive weeks. We found that CIH-induced genioglossus (GG) injury was correlated with mitochondrial dysfunction, reduction in the numbers of mitochondrias, impaired mitochondrial ultrastructure, and a reduction in type I fibers. Compared with the CIH group, impaired mitochondrial structure and function was significantly improved and a percentage of type I fiber was elevated in the CIH+AD group. Moreover, compared with the control group, the rats’ GG in the CIH group showed a significant decrease in phosphorylation of LKB1, AMPK, and PGC1-α, whereas there was significant rescue of such reduction in phosphorylation within the CIH+AD group.

**Conclusions:**

CIH exposure reduces mitochondrial biogenesis and impairs mitochondrial function in GG, while AD supplementation increases mitochondrial contents and alleviates CIH-induced mitochondrial dysfunction possibly through the AMPK pathway.

## Introduction

Recent studies have suggested that chronic intermittent hypoxia (CIH), a key pathological hallmark of obstructive sleep apnea hypoxia syndrome (OSAHS), is responsible for hypoadiponectinemia revealed in OSAHS patients [1], [2]. Further research also indicates that hypoadiponectinemia induced by CIH is associated with genioglossal dysfunction, which has been recognized as a crucial cause of OSAHS and could be improved by adiponectin (AD) supplementation [3]. However, the molecular mechanism of this process has not been fully elucidated.

AD, as an adipocyte-specific protein, plays an important role in the regulation of glucose and lipid metabolism, specifically inflammatory and oxidative stress [4]–[6]. Plasma AD levels are found to be decreased in OSAHS, obesity, type 2 diabetes, and insulin resistance [7], [8]. Supplementation of AD in contrast has been shown to increase mitochondria numbers and oxidative metabolism [9], [10]. Conversely, AD-deficient mice exhibited decreased mitochondrial number and increased mitochondrial dysfunction [11]. The mechanism behind these effects is related to AMP-activated protein kinase (AMPK) [9], [11]. Our previous study showed that CIH could cause ultrastructural degenerative changes and mitochondrial dysfunction in gengioglossus [3]. However, the exact mechanisms of mitochondrial injury have yet to be ascertained.

The aim of this study is to further investigate the mechanisms by which CIH induces mitochondrial injury of genioglossus, and whether AD supplementation can protect CIH-induced genioglossal mitochondrial injury and involved pathways.

## Materials and Methods

This study was approved by the Animal Ethic Committee of Nanjing Medical University.

### Animals

45 healthy male Wistar rats (aged 8 weeks) were bought from Shanghai Silake Ltd. Inc. of Animal Experiment. Rats were randomly distributed into three groups of 15. Rats in the control group were kept in a normoxic environment, rats in the CIH group were maintained under CIH condition for 35 days, and rats in the CIH+AD group were exposed to the same milieu as the CIH group with the addition of regularly administrated AD (R&D Inc., USA) via tail vein. Rats in the control group and CIH group were given saline through tail vein instantaneously. On day 35 of the experiment, the rats were anesthetized by using 1–2% isoflurane. After successful anesthesia, the rats had their abdominal cavitites quickly incised, blood drawn from the abdominal aorta followed by removal of the abdominal aorta. Subsequent acute hemorrhage resulted in rapid and non-violent exsanguination of the rats.

### CIH exposure

The exposure of CIH was performed as previously described [3]. During the different exposures, the rats were placed in 2.5-liter separately sealed spaces. Rats were allowed to move and obtain water and food freely in the sealed spaces. During the CIH exposure, each cycle of intermittent hypoxia lasted for 2 minuteswith an initial minute of hypoxic exposure followed by a minute of reoxygenation. During the hypoxic period, the O_2_ concentration in the CIH chambers was rapidly decreased to 5∼6% by prompt flushing with 99% N_2_. The nadir O_2_ lasted for 15∼20 s per the cycle. During the reoxygenation period, the O_2_ concentration was increased to 21% at maximum by rapidly filling the room with room airoxygen. Rats in the three groups were exposed to each condition daily for 8 daylight hours for 5 consecutive weeks. Rats in the control group and CIH group were given saline at the dose of 500 µl per time via tail vein, and rats in the CIH+AD group were administered AD through tail vein at the dosage of 10 µg per time, twice a week for 5 successive weeks.

### Observation of genioglossal ultrastructure

As soon as the rats were euthanized, their genioglossi were rapidly detached and sliced into small approximate 1.0 mm^3^ pieces at and fixed in 5% glutaraldehyde phosphate buffer at 4°C for 2 hours. The samples were hereafter postfixed in 1% Osmium tetroxide at 4°C for 1 hour, dehydrated in graded alcohol, and embedded in Epon at either a transverse or longitudinal direction. The samples were cut by the RMC/MTX ultramicrotome (Elexience), the sections (between 60 and 80 nm each) were transferred to copper grids, and observed using transmission electron microscope (JEOL, JEM-101, Japan). The analysis was carried out by Soft Imaging System.

### Western blot analysis

Cytoplasmic proteins were extracted from the GG, and immunoblots were implemented as previously described [12]. Protein homogenates were subjected to Western blot analysis with antibodies against phosphor-LKB1 (Cell Signaling Technology, USA), phosphor-AMPK (Cell Signaling Technology, USA), PGC1-α (Santa Cruz Biotechnology, USA), Troponin I (Santa Cruz Biotechnology, USA), Myosin chain 1 (Vectorlabs, USA) and GAPDH (Santa Cruz Biotechnology, USA),followed by incubation with a peroxidase-conjugated secondary antibody. The signals were detected with the electrochemiluminescence ECL system (Bio-Rad Laboratories Inc., USA) and quantification was carried out by measuring the densitometry of the signals with the Image J software.

### Quantitative real-time PCR

The mRNAs of genes involved in this study are listed in Table 1. Rats were anesthetized and the genioglossi was sectioned and frozen in liquid nitrogen. Isolation of total RNA was extracted from the GG with Trizol reagent (Takara Bio Inc., Japan). Quantitative detection of gene expression was carried out on a ABI 7900 Real Time PCR System (Applied Biosystems Inc., USA). Primer sequences (Table 1) were designed by Invitrogen (Shanghai, China). Quantities of all genes were normalized to the rat GAPDH. Values were relative to each expression in control group respectively.

**Table 1:** Primer sequences.

Gene(Rat)	forward	reverse
Hmox1	GTCAAGCACAGGGTGACAGA	CTGCAGCTCCTCAAACAGC
Cycs	GATGCCAACAAGAACAAAGGT	TGGGATTTTCCAAATACTCCAT
NQO1	AGCGCTTGACACTACGATCC	CAATCAGGGCTCTTCTCACC
Cox4i1	CACTGCGCTTGTGCTGAT	CGATCAAAGGTATGAGGGATG
OGG1	ATGGCTTCCCAAACCTTCAT	CAACTTCCTGAGGTGGGTCT
Ant1	GTAGGATGATGATGCAGTCTGG	CGTCCTTCATCTTTTGCAATC
Cs	GCACGCCAGTGCTTCTTC	CATGCTGCTGTCTGAAGGTC
Esrrα	CTTCCCTGCTGGTCCTCTG	CACCAGGGCGTTAACTGG
Nrf1	CCAAACCCACAGAGAACAGAA	TCCATGCATGAACTCCATCT
PGC1-a	GATGCCAACAAGAACAAAGGT	TCTGGGGTCAGAGGAAGAGA
MHC1	CACCAACAACCCCTACGATT	AGCACATCAAAGGCGCTATC
MHC2a	TCAAATCATCAGTGCCAACC	TGCCAAAGTGAATCCTGATG
MHC2x	AGAGGCCAAAAGGAAAGAGC	TCAGCATCAGCCAAGCTGT
MHC2b	CCAGTTGAACCATGCCAAC	TCTGAGAGCATCGTCCAGGT
GAPDH	GCAAGTTCAATGGCACAG	CATTTGATGTTAGCGGGAT

Hmox1, heme oxygenase; Cycs, cytochrome C; NQO1, NAD(P)H dehydro-genase-quinone-1; Cox4i1, cytochrome oxidase subunit IV isoform 1; OGG1, 8-oxoguanine DNA glycosylase; Ant1, Adenine nucleotide translocator 1; Cs, citrate synthase; Esrrα, estrogen-related receptorα; Nrf1, nuclear respiratory factor 1; PGC1-α, peroxisome proliferative activated receptor gamma coactivator 1-α; MHC, myosin heavy chain; GAPDH, glyceraldehyde-3-phosphate dehydrogenase.

### Histological analyses

Samples of the GG were frozen down in liquid nitrogen-cooled isopentane. Enzyme histochemical staining for cytochrome oxidase activity (COX) [13] and succinate dehydrogenase (SDH) activity [14] were performed as described. We assessed COX and SDH activities by enzyme histochemistry.

### Statistic analysis

Normal distributed data are expressed as mean ± SEM while non-normal distributed date are expressed as median ± interquartile. For normal distributed continuous variables, One-Way ANOVA was used, with a Newman-Keuls test to determine post hoc differences. For non-normal distributed variables, Kruskal-Wallis test was used with a post-hoc Dunn’s multiple comparison.

## Results

### Changes of mitochondrial biogenesis

**Figure 1:**
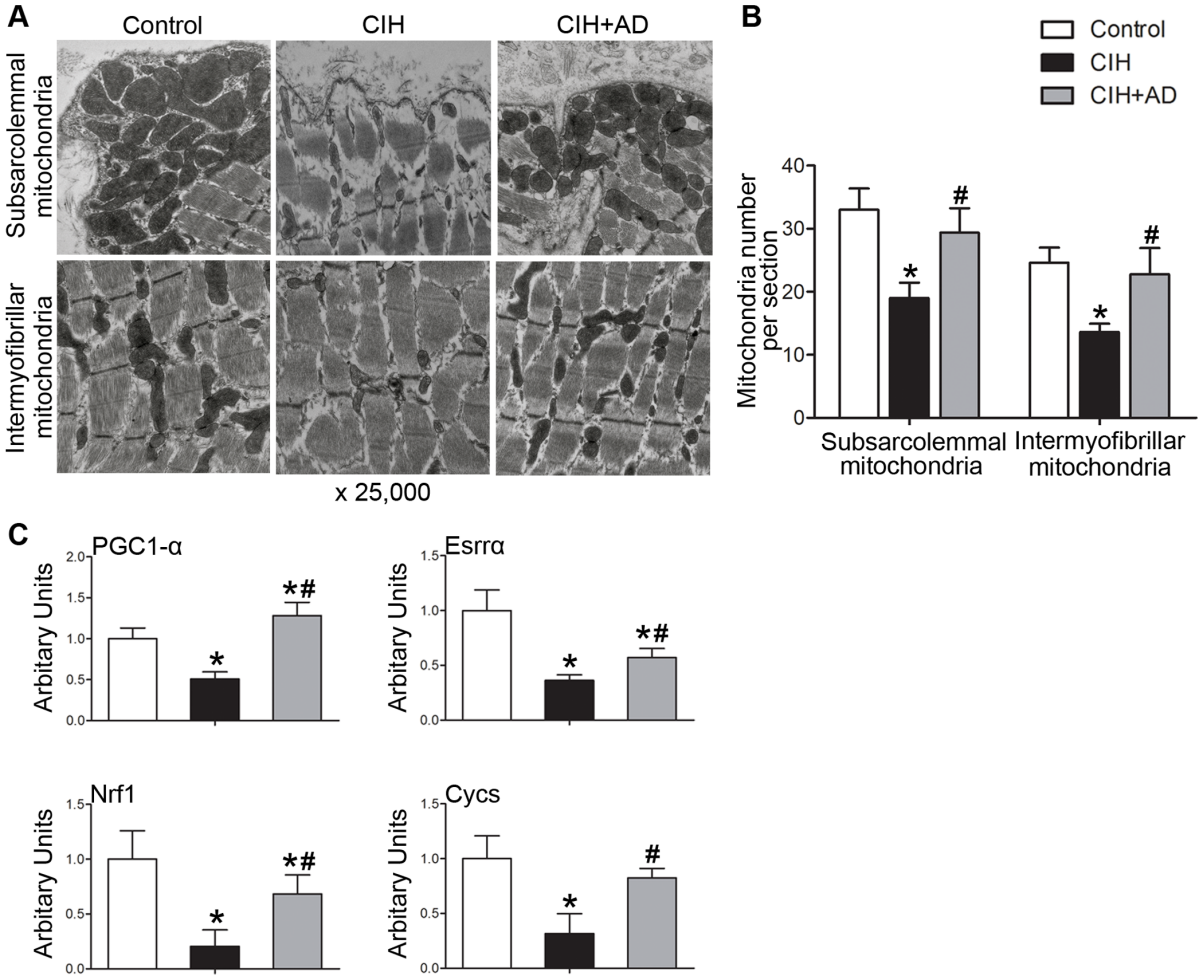
Changes of mitochondrial biogenesis in the three groups. (A) Mitochondrial density assessed by electron microscopy in the genioglossus muscle of rats from three groups. Original magnification, ×25,000. (B) Quantification of subsarcolemmal and intermyofibrillar mitochondria number per image area in the genioglossus muscle of rats from three groups (analysis of 5 images in 3 rats per group). *: P<0.05, CIH or CIH+AD compared with control, #: P<0.05, CIH+AD compared with CIH. (C) Levels of mRNAs implicated in mitochondrial biogenesis based on quantitative real-time RT-PCR performed on RNA isolated from genioglossus muscle from three groups (n = 6–8 per group). Results were normalized by the mean value for the control rats set to 1 unit.

As shown in Figure 1A and 1B, the amounts of both subsarcolemmal and intermyofibrillar mitochondria were lower in CIH group than those in both control group and CIH+AD group (p<0.05), with no significant differences between control group and CIH+AD group (p>0.05).

To ascertain the mechanisms associated with the reduction of mitochondrial density in the GG of the CIH group, we measured the mRNA levels of genes such as PGC1-α, estrogen-related α receptor (Esrrα), nuclear respiratory factor 1(Nrf1), and cytochrome C (Cycs) since they usually reflect the degree of mitochondrial biogenesis (Figure 1C). The mRNA levels of PGC1-α, Esrrα, Nrf1 and Cycs in GGi were all significantly lower in CIH group than both control group and CIH+AD group (p<0.05). Comparison between control and CIH+AD groups showed that a) no significant difference in Cycs levels; b) a statistically higher PGC1-α level in CIH+AD group; c) a significantly lower Esrrα and Nrf1 levels in CIH+AD group.

### Comparison of genioglossal mitochondrial ultrastructure

**Figure 2:**
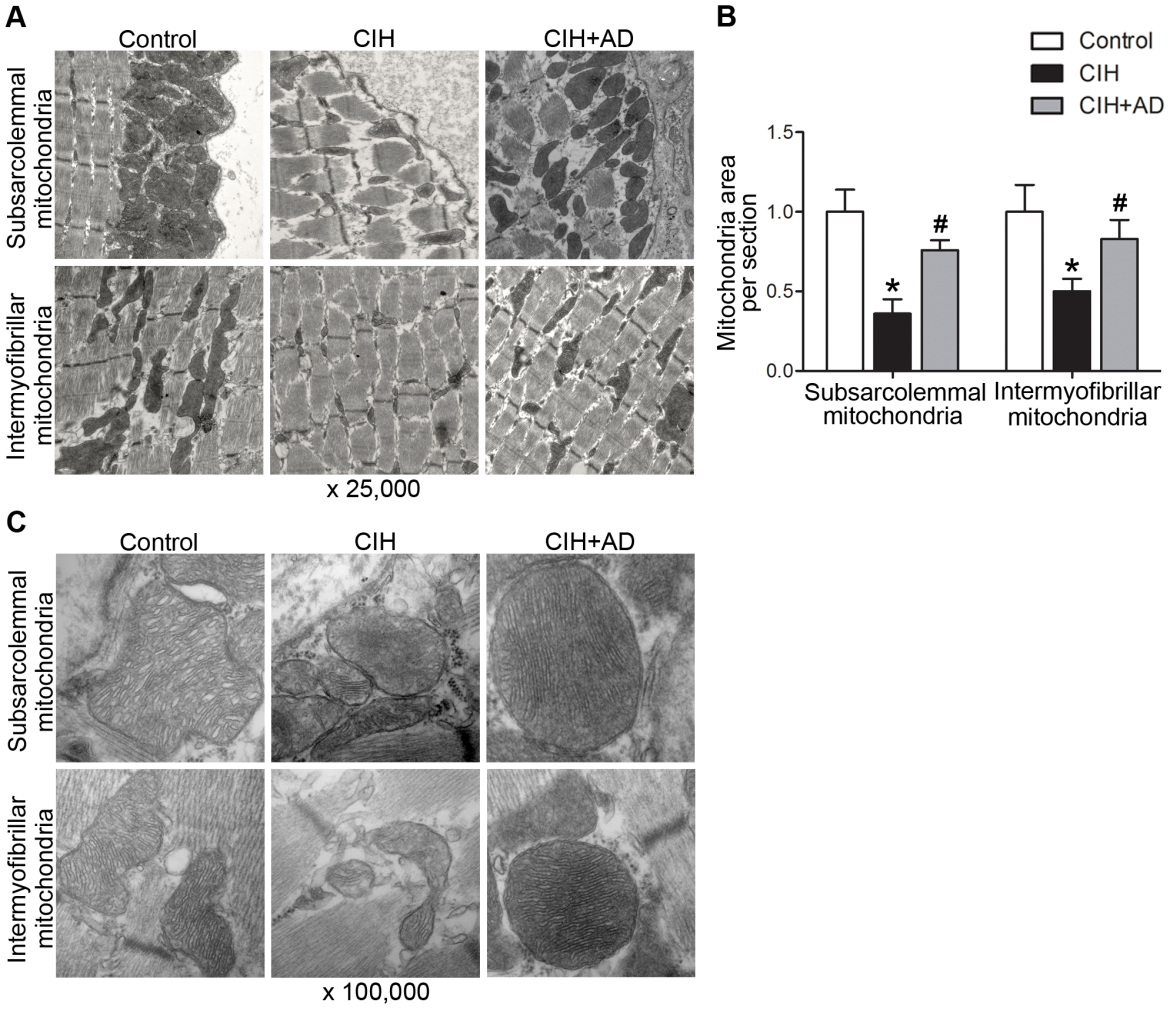
Alteration of mitochondrial ultrastructure in the genioglossus of the three groups. (A and C) Transmission electron microscopy images at original magnifications of ×25,000 (A) and ×100,000 (C) in subsarcolemmal and intermyofibrillar mitochondria from the genioglossus muscle of rats from three groups. (B) Quantification of subsarcolemmal and intermyofibrillar mitochondria area in the genioglossus muscle of rats from three groups (analysis of 5 images in 3 rats per group). *: P<0.05, CIH or CIH+AD compared with control, #: P<0.05, CIH+AD compared with CIH.

In addition to the reduction in mitochondrial density, marked alterations in mitochondrial morphology was also seen under transmission electron microscopy in the CIH group. Areas of both subsarcolemmal and intramyofibrillar mitochondria were notably lower in the CIH group than those in both control and CIH+AD group (Figures 2A and 2B). Swelling of mitochondria, a reduced electron density of the matrix, and an increased number of disarrayed cristae were shown only in the CIH group upon higher magnification (X100, 000) (Figure 2C).

### Comparison of mitochondrial function

**Figure 3:**
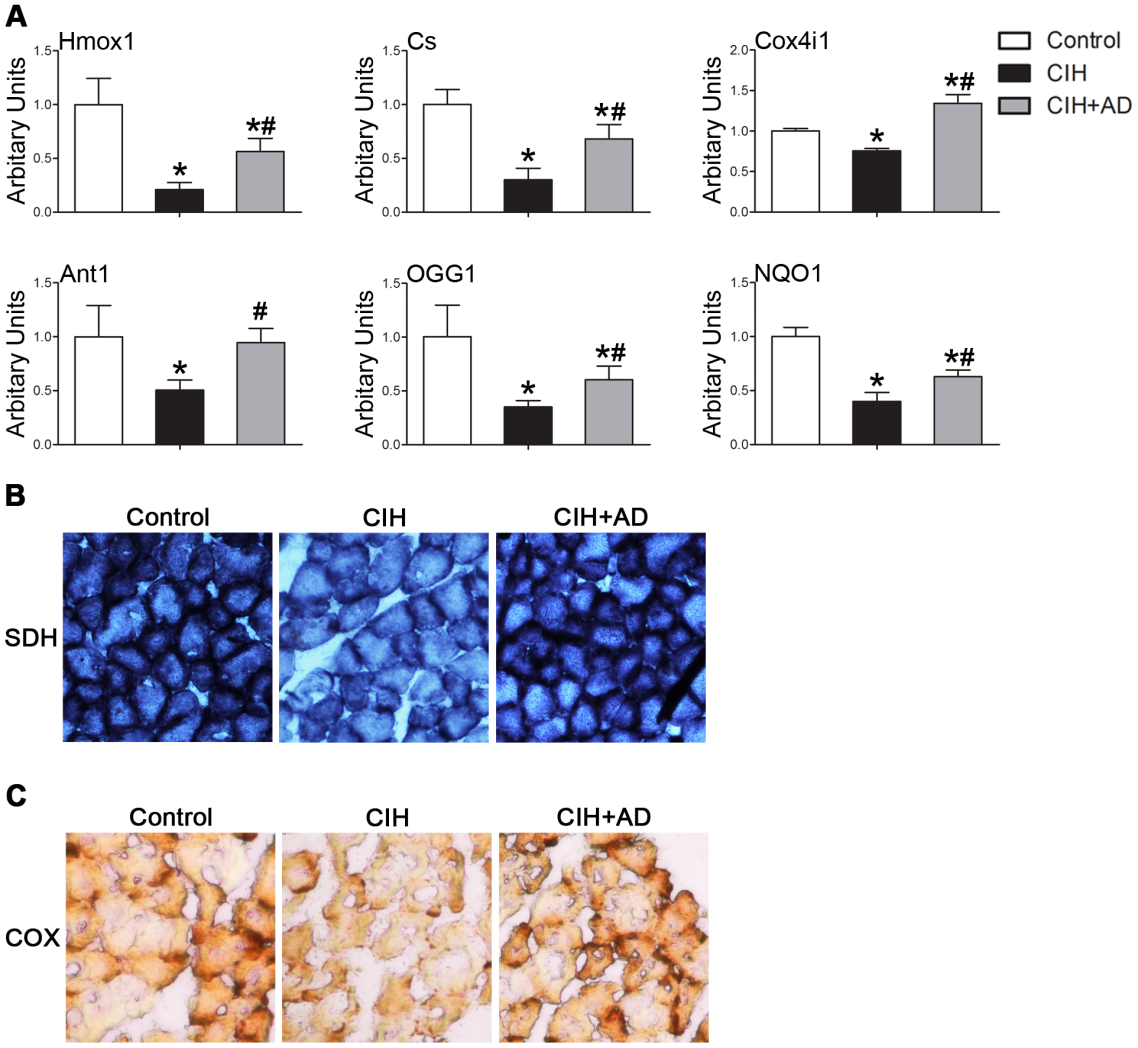
Changes of mitochondrial function in the three groups. (A) Levels of mRNAs implicated in mitochondrial function based on quantitative real-time RT-PCR performed on RNA isolated from the genioglossus muscle of three groups (n  =  6–8 per group). Results were normalized by the mean value for the control rats set to 1 unit. (B) Succinate dehydrogenase (SDH) activity staining was performed on sections of the genioglossus muscle of three groups (n  =  6–8 per group). (C) Cox activity staining was performed on sections of the genioglossus muscle of three groups (n  =  6–8 per group). *: P<0.05, CIH or CIH+AD compared with control, #: P<0.05, CIH+AD compared with CIH.

To detect whether changes in ultrastructure and mitochondrial density resulted in genioglossal mitochondrial dysfunction in the CIH group, we measured the expression of genes associated with mitochondrial function including the TCA cycle, OXPHOS, electron transport chain, and mitochondrial membrane organization with quantitative RT-PCR. The mRNA levels of genes related to mitochondrial function, such as heme oxygenase (Hmox1), citrate synthase (Cs), cytochrome oxidase subunit IV isoform 1 (Cox4i1), Adenine nucleotide translocator 1 (Ant1), 8-oxoguanine DNA glycosylase (OGG1), and NAD(P)H dehydro-genase-quinone-1 (NQO1) were all markedly lower in the genioglossus of CIH group than both control and CIH+AD groups (Figure 3A). Mitochondrial function was evaluated by estimating the enzymatic activities of succinate dehydrogenase (SDH) and Cox to further verify the changes in mitochondrial functions. Stained genioglossi sections demonstrated a lower number of Cox- and SDH-positive muscle fibres and a reduced intensity of SDH and Cox staining in CIH group (Figures 3B and 3C). In addition, the mRNA levels of Hmox1, Cs, Cox4i1, Ant1, OGG1, and NQO1 were remarkably higher in the GG of the CIH+AD group than those in the CIH group (Figure 3A). Stained genioglossi sections also showed a higher number of SDH- and Cox-positive muscle fibres and an increased intensity of SDH and Cox staining in the CIH+AD group than those in CIH group (Figures 3B and 3C).

### Comparison of genioglossal fiber-types

**Figure 4:**
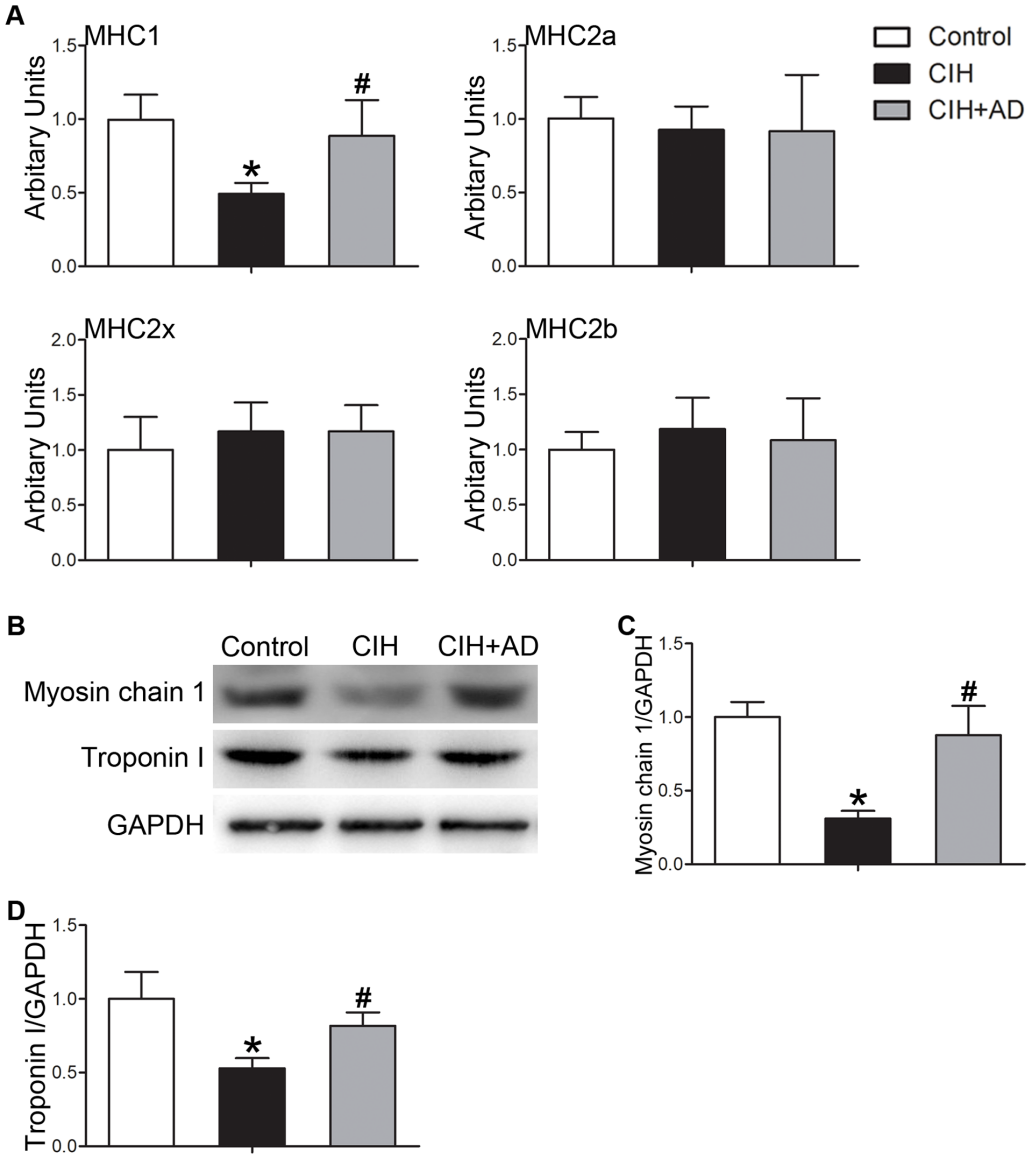
Genioglossus fiber-type changes in three groups. (A) Levels of MHC1, 2a, 2x and 2b mRNA based on quantitative real-time RT-PCR performed on RNA isolated from the genioglossus muscle homogenates in three groups. (n = 6–8 per group). (B) Western blot analysis of myosin chain 1, troponin I and GAPDH using the genioglossus muscle homogenates in three groups. (C and D) Densitometric analysis of Western blots (n = 5 per group). Results were normalized by the mean value for the control rats set to 1 unit. *: P<0.05, CIH or CIH+AD compared with control, #: P<0.05, CIH+AD compared with CIH.

As shown in Figure 4A, the mRNA levels of MHC1 was remarkably lower in the CIH group than those in control and CIH+AD groups (p<0.05). However, the levels of 2a, 2x, and 2b mRNA were not different among the three groups (p>0.05). The expression of markers of type I fibers such as myosin chain 1 and troponin I was reduced in CIH groups, compared with the control and CIH+AD groups (Figures 4B, 4C, and 4D).

### Adiponectin-mediated signaling

**Figure 5:**
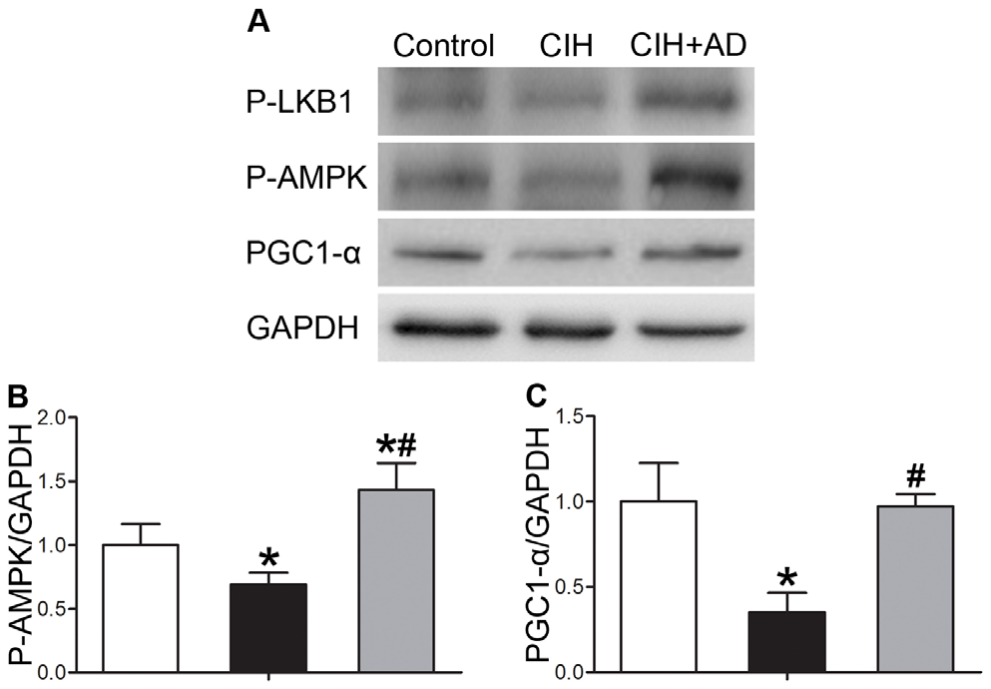
Adiponectin-mediated signaling. (A)Western blot analysis of P-LKB1, P-AMPK, PGC1-α and GAPDH using the genioglossus muscle homogenates in three groups. (B and C) Densitometric analysis of Western blots (n = 5 per group). Results were normalized by the mean value for the control rats set to 1 unit. *: P<0.05, CIH or CIH+AD compared with control, #: P<0.05, CIH+AD compared with CIH.

AMPK, as a pathway, is allosterically activated by alterations in the ratio of AMP/ATP and covalently phosphorylated at Thr172 by upstream kinase AMPK kinase. Adiponectin can activate the LKB1/AMPK/PGC1-α signaling pathway, which has impact on mitochondrial contents and function [9]. Therefore, we examined the effect of CIH and CIH+AD on AMPK activation. As shown in Figure 5A, 5B, and 5C, phosphorylation of LKB1, AMPK, and PGC1-α was significantly decreased in the CIH group, compared with the control group (p<0.05). Such weakened phosphorylation of LKB1, AMPK, and PGC1-α in the CIH group was so significantly increased in CIH+AD group that there was no statistical difference in degree of phosphorylation between control and CIH+AD groups.

## Discussion

Upper airway dilator muscles (UADM) play a crucial role in the regulation of airway patency [15], [16]. Dysfunction of UADM renders the organism susceptible to upper airway collapse. Impairment of UADM’s structure and function occur in patients with OSAHS, and functional injury of UADM results in collapsibility of upper airway causing CIH in patients with OSAHS [15], [17]. In our current study, we focused on the mitochondrion of the genioglossus (GG) because GG is one of the most important UADM [18], [19] and the mechanisms driving genioglossal dysfunction might be related to impaired energy metabolism. Abnormal function of the GG can cause increased upper airway resistance and even induce its collapse [19]. Recent studies shown that the impairment of the GG’s structure and function may be in association with abnormal energy metabolism in GG of OSAHS patients [15], [17], [20]. However, the specific mechanism is not yet clarified. Mitochondrium, as the central base for cellular energy metabolism, can better reflect the conditions of energy production and transmission within cells [21]. Here, we explore the structure, quantity, and function of genioglossal mitochondria in rats exposed to CIH.

PGC1-α is a key regulator of mitochondrial biogenesis [22]–[24]. Our study found that under CIH conditions, PGC1-α mRNA and PGC1-α protein levels were significantly reduced. Likewise, the mRNA levels of other genes associated with mitochondrial biogenesis, such as Esrrα, Nrf1 and Cycs, were all significantly lower in the CIH group. Furthermore, we observed the structure of the GG mitochondria under electron microscopy and found that the mitochondria appeared swollen, with fewer cristae and lower matrix density, severely destroyed inner and outer membrane integrity, and significantly reduced mitochondrial area in the rats of the CIH group. These mitochondrial abnormalities implicate CIH as a driving force in the impairment of GG mitochondrial content and structure. It has been reported that intermittent hypoxia could also cause brain mitochondrial dysfunction [25]. Shin-Da Lee et al found that CIH additionally weakened the mitochondrial oxidative capacity of cardiomyocytes [26]. In our study, we found that the expression levels of several genes involved in mitochondrial function were significantly reduced in the CIH group. In addition, the stained genioglossal sections revealed the decrease in the enzymatic activities of Cox and SDH. Moreover, CIH resulted in a remarkable decrease of genioglossal type I fibers [27], [28], which might explain why the proportion of upper airway dilator muscle type I fibers was significantly reduced in OSAHS patients. Collectively, the data suggest that CIH induces mitochondrial dysfunction of the GG. The mitochondrial dysfunction and reduced type I muscle fibers are likely to contribute to pathogenesis of contractile dysfunction of GG, as affirmed by other studies [15], [29].

Previous studies have demonstrated that blood AD levels are decreased in OSAHS patients [1], [3], [15]. However, the relation between AD and mitochondrial function of the GG has not been fully elucidated. The current study revealed that AD supplementation could reverse hypoadiponectinemia and mitochondrial dysfunction associated with CIH [3]. Anthony E. and other studies have shown that AD could increase PGC1-α expression to promote mitochondrial content in skeletal muscle cells [9], [11]. Consistent with our results, AD supplementation could promote GG mitochondrial synthesis even under CIH conditions. Moreover, after AD intervention, CIH-induced mitochondrial ultrastructural damage was significantly alleviated. Mitochondrial ultrastructural damage can substantially interrupt mitochondrial oxidative phosphorylation, and thereby decrease mitochondrial ATP generation. In mice with AD gene knockdown, reduced mitochondrial content and mitochondrial enzyme activity in skeletal muscle was reported, while AD treatment could improve mitochondrial biogenesis and mitochondrial enzyme activity [9]. In our study, we clearly demonstrated that exogenous AD supplementation could enhance the activity of cytochrome oxidase and succinate dehydrogenase, and improve the mRNA levels of Hmox1, Cs, Cox4i1, Ant1, OGG1 and of NQO1. Anthony E. et al found that AD could additionally switch type II fibers into type I fibers [9]. Our finding also showed that AD could significantly increase the expression level of PGC1-α. Overexpression of PGC1-α in muscle can result in a remarkable increase in the expression of troponin-1 protein and type I fiber [30]. In accordance with previous studies, we found that AD treatment increased the troponin-1 protein expression and type I muscle fiber content. These findings indicate that AD could improve the CIH-induced mitochondrial dysfunction and increase the type I muscle fiber content.

The AMP-activated protein kinase (AMPK) is a conserved sensor of cellular energy state, and it is reported that the AMPK pathway plays a pivotal role in regulating energy balance [31], [32]. Chronic activation of AMP kinase can result in enhanced mitochondrial biogenesis and oxidative capacity [33]. AD can stimulate glucose utilization and fatty acid oxidation by activating AMP-activated protein kinase [11], [34]. Our study demonstrated that CIH partially inhibited the expression of AMPK pathway, whereas AD supplementation could imminently elevate the expression of LKB1 (AMPKK)/AMPK/PGC1-α. Therefore, it is implied that AD rescues CIH-induced reductions in mitochondrial synthesis and oxidative capability through the AMPK pathway.

In spite of the fact that CIH is a consequence of apneas and hypopneas and cannot be the primum movens of genioglossal dysfunction, our results seemed to be able to explain that the OSAHS frequently becomes more severe during aging and when metabolic and cardiovascular comorbidities are associated with OSAHS.

It is also possible that CIH induced hypoadiponectinemia can further lead to reduced mitochondrial biogenesis and impaired mitochondrial function in genioglossus through insulin resistance, because the association between AD and insulin resistance is evident in clinical studies by showing that hypoadiponectinemia is a risk factor for insulin resistance represented by HOMA index [35], [36]. It is unfortunate that the HOMA index was not tested in the current study. Since insulin resistance is a metabolic condition characterized by an impaired ability of plasma insulin to regulate glucose homeostasis in target tissue, such as skeleton muscle or adipose tissue [37], further studies are needed to investigate the relationship between AD and insulin resistance at a mitochondrial level within genioglossal tissue.

In conclusion, the present study demonstrated a) CIH could reduce mitochondrial biogenesis and impair mitochondrial function; b) AD supplementation could increase mitochondrial contents and alleviate mitochondrial dysfunction induced by CIH; c) AD might mediate the mitochondrial contents and function through the AMPK pathway. Our data strongly suggest that AD supplementation is a feasible novel therapeutic approach to prevent CIH-induced self-aggravation of genioglossal dysfunction in human OSAHS. Further clinical studies are therefore needed.
